# Prophylactic treatment of flea-infested cats with an imidacloprid/flumethrin collar to forestall infection with *Dipylidium caninum*

**DOI:** 10.1186/1756-3305-5-151

**Published:** 2012-07-27

**Authors:** Josephus J Fourie, Dionne Crafford, Ivan G Horak, Dorothee Stanneck

**Affiliations:** 1 ClinVet International, P.O. Box 11186, Universitas, 9321, South Africa; 2 Department of Zoology and Entomology, University of the Free State, Bloemfontein, 9301, South Africa; 3 Department of Veterinary Tropical Diseases, Faculty of Veterinary Science, University of Pretoria, Onderstepoort, 0110, South Africa; 4 Bayer Animal Health GmbH, D-51368, Leverkusen, Germany

**Keywords:** Imidacloprid, Flumethrin, Collars, Prophylaxis, Fleas, *Dipylidium caninum*, Cats

## Abstract

**Background:**

The objective of the study was to determine the sustained effectiveness of 10% imidacloprid (w/w) and 4.5% flumethrin (w/w) incorporated in a slow-release matrix collar in preventing *Dipylidium caninum* infection in cats following repeated laboratory-infestations with fleas infected with metacestodes.

**Methods:**

Efficacy against infection with *D. caninum* was evaluated by infesting 16 cats with the flea *Ctenocephalides felis felis* infected with metacestodes of the tapeworm. Medicated collars were fitted to 8 of the cats and infestation of each cat with 200 fleas from a suitably infected batch commenced 7 days later and continued at weekly intervals until Day 28. Efficacy against fleas was evaluated 24 h after each infestation. Infection of the cats with *D. caninum* was verified by daily examination of the cats’ faeces and immediate surroundings for proglottids from Day 21 to Day 60. Calculation of the prophylactic effectiveness of the collars in preventing infection of the cats with *D. caninum* was based on the difference in the geometric mean number of scoleces recovered from the gastrointestinal tracts of collared compared to untreated cats at necropsy on Day 61.

**Results:**

Efficacy of the collars against infestation of the cats with fleas was 99.9% on Day 7 and 100% at each subsequent weekly assessment. Infection of the fleas with metacestodes was ≥40% in 7 to 13 day old fleas, but progressively decreased thereafter. At necropsy all the control cats were infected with *D. caninum* and harboured between 19 and 346 scoleces with a geometric mean of 58.3. A single treated cat was infected and harboured 2 scoleces. Effective prevention of infection with *D. caninum*, based on a comparison of the geometric mean numbers of scoleces recovered from control and treated cats, was 99.7%.

**Conclusion:**

The insecticidal components of the medicated collars are capable of rapidly eliminating newly-acquired infestations of fleas that are infected with the metacestodes of *D. caninum*, thus preventing infection with the cestode in collared cats.

## Background

The cat flea, *Ctenocephalides felis felis* is widespread throughout most regions of the world, and infests both cats and dogs [1,2]. The effective control of *C. felis felis* should not only alleviate the immediate distress caused to its hosts, such as itching, hair loss and skin lesions caused by continuous grooming, but also prevent the development of flea allergy dermatitis provoked by the saliva of feeding fleas [3]. *C. felis felis* is also a host of the metacestode stage of the tapeworm *Dipylidium caninum*[4]. The adults of this cestode infect cats and dogs should they swallow infected fleas while grooming.

Infection of cats and dogs with the cestode *D. caninum* appears to be a world-wide phenomenon. In Hawaii 81% of 107 stray or abandoned cats were infected, in Australia 19% of 400 cats, and in South Africa 23% of 1 502 cats examined [5-7]. Prevalences of 12% and higher have also been reported in cats in England, Scotland, Denmark, Iraq and Nigeria [8]. A high prevalence of infection may also be accompanied by a high intensity of infection and mean burdens of 38.4 *D. caninum* have been recorded in naturally infested juvenile cats and 25.9 in adult cats in South Africa [7]. These comparatively large burdens in naturally infected cats and the large proportion of animals infested in several regions of the world are an indication of the extent of the problem.

It is generally accepted that infection with *D. caninum* produces few if any clinical signs in cats [8]. However, infection of pets is usually a cause of considerable distress and often embarrassment to their owners. More importantly, humans, and particularly small children, who come into close contact with family pets, and hence the fleas that infest them, may also become infected with this cestode [9]. There appears to be no impediment to the development of *D. caninum* in young children, and a 6-month old infant passed 13 intact tapeworms, of which the longest was approximately 40 cm, and several short chains of proglottids, in a stool collected the day after treatment [10].

The effectiveness of a spot-on formulation of imidacloprid (Advantage^©^) against fleas on cats has been demonstrated by Genchi *et al*. [3], who also documented a significant decrease in the clinical signs associated with flea allergy dermatitis in the treated animals. In laboratory studies imidacloprid in combination with flumethrin in a slow-release matrix collar formulation (Seresto^©^), forestalled the development of flea allergy dermatitis in collared cats repeatedly infested with *C. felis felis* compared to similarly infested untreated cats [11]. The possibility has also been mooted that because newly acquired infestations of fleas are killed within hours of accessing an imidacloprid/flumethrin collared cat they are less likely to be ingested by the cat while grooming [11]. Should these fleas be infected with the metacestodes of *D. caninum* the life cycle of the tapeworm will be interrupted and the chances of infection becoming established in collared cats considerably reduced.

The aims of the present investigation were to evaluate the prophylactic effectiveness of treatment with the slow-release matrix collar formulation of 10% imidacloprid (w/w) and 4.5% flumethrin (w/w) against fleas infected with metacestodes of *D. caninum* and thus prevent infection of cats with the cestode.

## Methods

The study was conducted in South Africa and was a parallel group-designed, randomised, unicentre, controlled efficacy study on two groups of cats each consisting of eight animals. The cats enrolled in the study were housed indoors in an environmentally controlled unit. Within this unit the cats in the treated and control groups were housed in separate rooms and each cat was individually confined to a stainless steel housing cage (70 x 60 x 75 cm). Each room had its own air conditioning system and temperature was maintained at ~20°C ± 4°C, with a 12 h light and dark cycle. The accommodation was in compliance with the requirements of the South African National Standard (SANS 10386:2008. The care and use of animals for scientific purposes). The cats were fed once daily with a commercially available cat feed according to the food manufacturer’s recommendation. Food and water were provided in stainless steel bowls and the water was replenished at least twice daily. The cats were maintained and handled with due regard for their welfare, and were acclimatized to the caged environment for seven days prior to the commencement of the study. Approval for conduct of the study (including experimental infection) was subject to ethical approval by the ClinVet Animal Ethics Committee (CAEC).

Twenty short-haired, domestic, mixed-breed cats ranging in age from sub-adult to adult were enrolled in the study. These cats had not been treated with an acaricide, or insecticide, or a compound with an insect growth-regulating activity during the previous 12 weeks and were not infected with *D. caninum*. As an added precaution all cats were de-wormed with Triworm-C (praziquantal 20 mg/pyrantel pamoate 230 mg; Cipla-Vet) prior to the commencement of the study. Six days prior to the commencement of the study the cats were all infested with 100 *C. felis felis* and the subsequent pre-treatment flea count of each animal was used for ranking and group allocation. The four cats with the lowest flea counts were excluded from the remainder of the study. The remaining 16 cats were ranked in descending order of their individual pre-treatment flea counts, and their IDs were used to break ties. The cats were then blocked into blocks of two animals each, and within each block cats were randomly allocated to two groups. The 16 cats included in the study weighed between 2.53 and 4.96 kg and their hair length varied between 17.75 and 33.73 mm.

On Day 0 the medicated collars were fitted to the necks of the cats allocated to the treatment group. Each collar was adjusted by means of the buckle to achieve a comfortable fit and any excess was cut off approximately 2 cm beyond the retaining loop. All collars were marked with the cat’s ID number so that in case a collar was accidentally dislodged, it could easily be identified and immediately re-applied. At pre-determined time intervals on the day that the collars were fitted all animals were carefully observed for adverse signs that could be ascribed to the collars or to the active ingredients that they contained.

A laboratory-bred strain of *C. felis felis*, originating from Hannover University, Germany, and routinely fed on cats in South Africa for at least 3 years, was used throughout the study. The fleas were infected with metacestodes of a South African strain of *D. caninum* by incubating thousands of flea eggs and the ensuing larvae on flea-rearing medium mixed with proglottids and eggs of the tapeworm at temperatures ranging from 24 to 28.5°C. The adult fleas that developed from the larvae were used for all post-treatment infestations of the cats. Prior to each infestation the prevalence and mean intensity of infection with metacestodes was determined by microscopic dissection of 100 fleas from a batch of fleas that had been exposed to *D. caninum* eggs. At the same time the degree of development of the metacestodes was determined. The age of the fleas examined was measured as days post-pupation. However, age was at best only approximate since the fleas pupated over a period of two to three days. Each cat was infested with 200 fleas from a suitably infected batch on the days listed in Table 1. The fleas used for infestation were unfed and of mixed sex and were not released on the cats in the vicinity of the collars. Cats were restrained by hand during infestation with fleas and during flea recovery.

**Table 1:** **Design of a study aimed at determining the effectiveness of imidacloprid/flumethrin collars in preventing*Dipylidium caninum*infection in cats repeatedly infested with infected fleas**

**Study day**	**Activity**
**-7 to - 1**	Acclimatization to cage environment
**- 6**	Infestation with non-infected fleas
**- 5**	Flea counts, 4 cats with lowest counts excluded from remainder of study
**- 2**	Ranking and allocation to 2 groups of 8 cats each
**0**	Imidacloprid/flumethrin collars fitted to treated group
**7**	Infestation with 200, infected* fleas**
**8**	Flea counts and re-infestation with the fleas that had been counted
**14**	Infestation with 200, infected* fleas**
**15**	Flea counts and re-infestation with the fleas that had been counted
**21**	Infestation with 200, infected* fleas**; daily examination of faeces for expelled proglottids commences
**22**	Flea counts and re-infestation with the fleas that had been counted
**28**	Infestation with 200, infected* fleas**
**29**	Flea counts and re-infestation with the fleas that had been counted
**60**	Daily examination of faeces for expelled proglottids ceases
**61**	Necropsy and collection and counting of scoleces

* The larval stages of these fleas were exposed to proglottids and eggs of *D. caninum*.

** Fleas approximately 10–14 days after pupation were used for infestation.

On each assessment day the fleas on the cats were collected by using a fine-toothed flea comb. Different combs were used between groups and within groups combs were washed between cats. Combing was performed by several strokes of the comb over each part of the cat’s skin surface, each time in the same direction and following the lie of the hair coat. Movement, from one part of the animal’s body to the next, was via strokes overlapping each other, so that no part of the skin surface was missed. After the completion of combing, the whole procedure was repeated so that all areas were combed at least twice. If necessary a third combing was performed until no live fleas were found. After all the fleas recovered from a particular cat had been counted, the live fleas in the collection were put back onto the same cat. The counting of fleas was not blinded since the control cats were not fitted with placebo collars, thus making blinding impossible.

Efficacy against *C. felis felis* was calculated as follows:

Efficacy (%) = 100 x (m_c_ – m_t_) / m_c_, where mc geometric mean number of live fleas on cats in the untreated control group; mt geometric mean number of live fleas on cats in the treated group.; mc, geometric mean number of D. caninum scoleces recovered from the untreated control group of cats; mt, geometric mean number of D. caninum scoleces recovered from the group of cats fitted with medicated collars.

From Day 21 to Day 60 the cats were observed daily to detect the presence of expelled proglottids. This involved visual, macroscopic examination of fresh faeces, the anal and perineal regions of the animals, their hair and their cages. If no proglottids were found, freshly excreted faeces were washed through steel-mesh sieves with an aperture size of 0.3 mm. The residues in the sieves were collected and suspended in a small amount of water, which was then examined macroscopically for the presence of proglottids. All proglottids or worm fragments were examined microscopically to ensure that they were those of *D. caninum*. Once proglottids had been found in the faeces of a cat on two separate occasions no further faecal or other examinations for proglottids were conducted for that cat.

All the cats were euthanazed on Day 61 by intravenous injection of Euthapent^TM^ (sodium pentobarbitone 200 mg/mL; Kyron laboratories) at an approximate dose of 1 ml/kg. Food was removed from the cages during the afternoon prior to euthanasia in order to reduce the volume of ingesta in the gastrointestinal tract at necropsy. At necropsy a ligature was applied at the ileo-caecal junction between the small and large intestines and the digestive tract from the stomach to the rectum was removed from the abdominal cavity. The small intestine, including the stomach, and the large intestines were treated separately. They were carefully cut open and their contents flushed with water, and their mucosa thoroughly scraped. All material that had been flushed, washed or scraped from the small intestines and stomach were then washed over a sieve with 0.15 mm apertures. The contents of the large intestines and their mucosal scrapings were washed over sieves with 0.30 mm aperture sizes. The residues in the sieves were collected and preserved with formalin in labelled bottles. The bottles were coded for each cat in order to blind the counting of *D. caninum* scoleces.

The effectiveness of the imidacloprid/flumethrin collars in the prevention of *D. caninum* infection was based on the difference between the geometric mean numbers of scoleces recovered from the control and treated groups of cats.

Prophylactic effectiveness against infection with *D. caninum* by infected fleas was calculated as follows:

Prophylactic effectiveness (%) = 100 x (m_c_ – m_t_) / m_c_, where mc geometric mean number of live fleas on cats in the untreated control group; mt, geometric mean number of live fleas on cats in the treated group.; mc, geometric mean number of D. caninum scoleces recovered from the untreated control group of cats; mt, geometric mean number of D. caninum scoleces recovered from the group of cats fitted with medicated collars.

## Results

The geometric mean flea counts of the two groups of cats on each of the assessment days and the efficacy of the imidacloprid/flumethrin collars against fleas are summarized in Table 2. The mean flea counts of the untreated control group of cats ranged from 62.5 to 76.8, indicating a robust flea challenge on each of the days on which infestation was applied. The mean flea counts of the group of cats fitted with medicated collars differed significantly (p < 0.05) from those of the untreated control group of cats on all post-treatment assessment days. The efficacy of the collars against infestation with *C. felis felis* was ≥99.9% for the 28-day duration of that part of the study devoted to fleas.

**Table 2:** **Efficacy of an imidacloprid/flumethrin collar applied on Study Day 0 against*Ctenocephalides felis felis*on repeatedly infested cats**

**Study day**	**Geometric mean number of fleas recovered**		**Efficacy (%)**
	**Untreated control cats**	Collared cats*	
**8**	76.8	0.1	99.9
**15**	74.5	0	100
**22**	62.5	0	100
**29**	73.8	0	100

* The flea counts of the collared group of cats differed statistically significantly (p < 0.05) from those of the untreated control group of cats on all post-treatment assessment days.

In adult fleas the prevalence of infection with metacesodes decreased progressively from ≥40% (fleas 7 to 13 days post-pupation) to 2% (fleas 25 days post-pupation) and to zero (fleas 29 days post-pupation) (Table 3). The average intensity of infection remained between 2.40 and 4.95 in fleas aged up to 16 days post-pupation and decreased thereafter (Table 3).

**Table 3:** **Development, prevalence and mean intensity of infection of*Dipylidium caninum*metacestodes in*Ctenocephalides felis felis***

**Fleas**		**Number of metacestodes**					**Infection**	
**Days post-pupation**	**No. examined**	**Position of rostellar hooks**				**Total**	**Prevalence (%)**	**Average intensity in infected fleas**
		**None**^**1**^	**Scattered**^**2**^	**Organizing**^**3**^	**Organized**^**4, 5**^			
**7**	100	161	0	0	0	161	43	3.74
**10**	100	49	148	1	0	198	40	4.95
**13**	100	19	81	1	0	101	42	2.40
**16**	100	15	45	3	2	65	21	3.10
**19**	100	0	12	8	1	21	12	1.75
**22**	100	0	5	3	1	9	5	1.80
**25**	100	0	1	0	1	2	2	1.00
**29**	100	0	0	0	0	0	0	0

^1^ No rostellar hooklest present (non-infective).

^2^ Hooklets visible (non-infective).

^3^ Some scattered hooklets still visible but starting to migrate to a single point (non-infective).

^4^ Hooklets organizing into characteristic circle/rosette shape (possibly infective), or ^5^ rostellum with organized hooklets clearly visible (infective).

**Figure 1:**
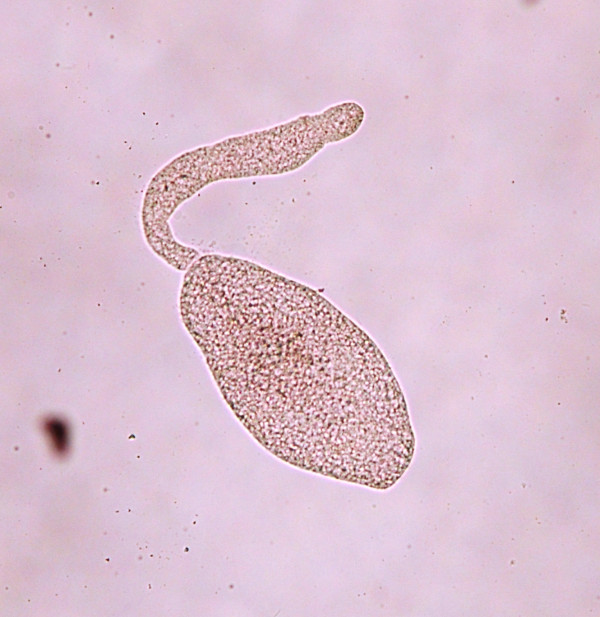
Developmental stage of a metacestode without hooklets.

**Figure 2:**
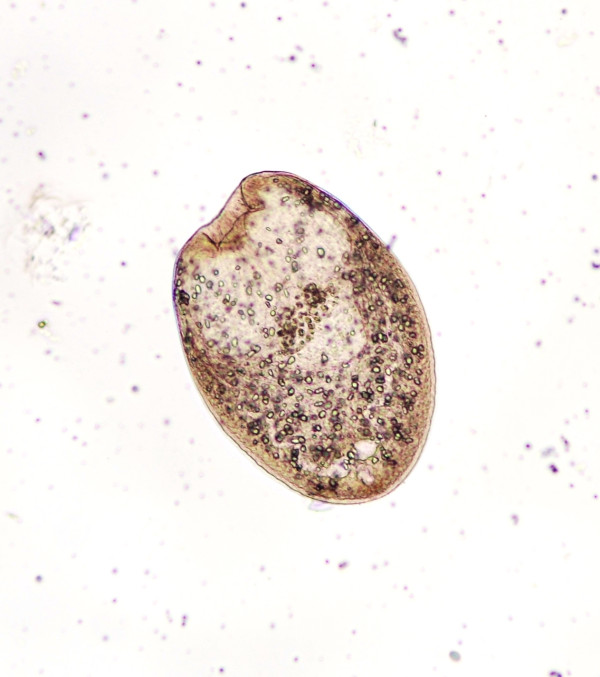
Developmental stage of a metacestode with scattered rostellar hooklets.

**Figure 3:**
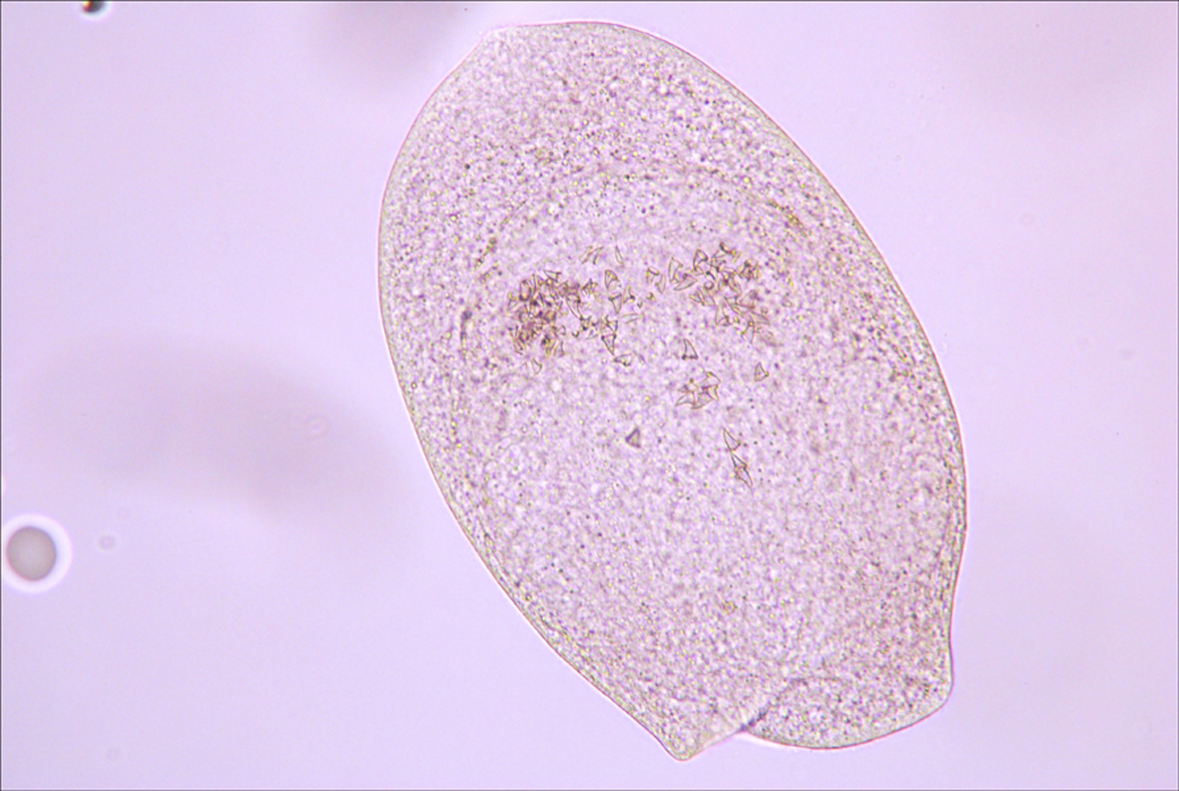
**Developmental stage of a metacestode with hooklets starting to become organized**.

**Figure 4:**
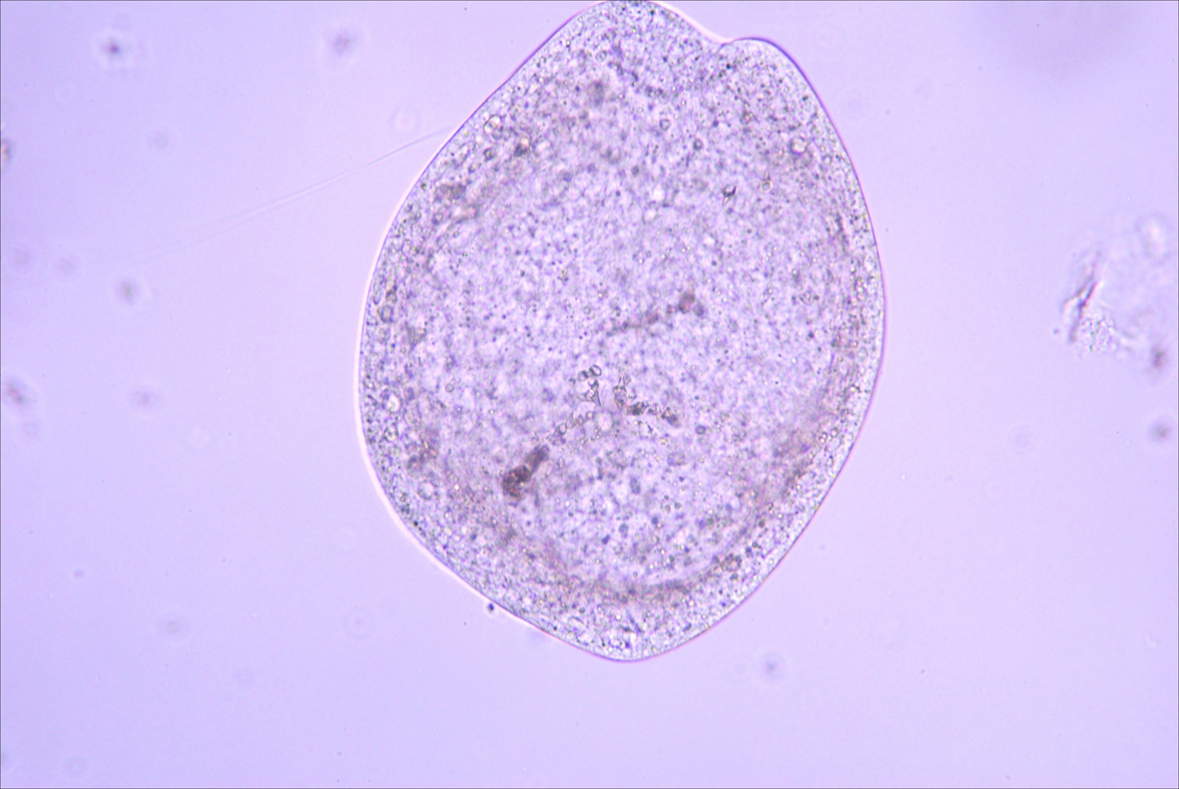
**Developmental stage of a metacestode with well organized hooklets**.

**Figure 5:**
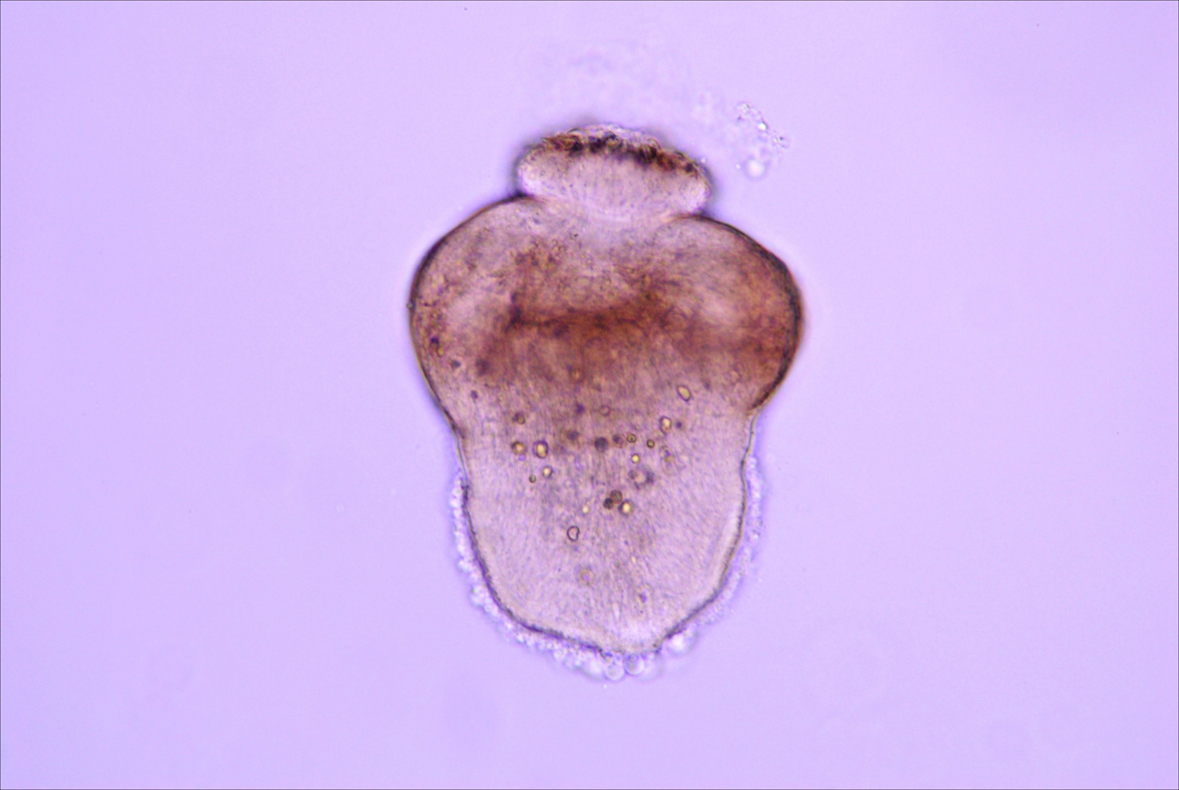
**Developmental stage of a metacestode with completely organized hooklets and everted rostellum**.

Metacestodes gradually matured in fleas older than 13 days post-pupation as judged by the development and arrangement of rostellar hooks (Figures 1, 2, 3, 4 and 5). The first metacestodes with an organized pattern of rostellar hooks were seen in fleas 16 days post-pupation (Table 3). The fleas used for infestation were mostly aged between 10 and 14 days post-pupation. Thus both prevalence and mean intensity of infection were reasonably high and the development of metacestodes fairly advanced, implying that the metacestodes in the fleas would require only a short period of time on the cat hosts before becoming infective.

The time between fitting the collars and detection of *D. caninum* proglottids in the cats’ faeces or in their immediate surroundings are summarized in Table 4. All the cats in the untreated control group and one cat in the collared group shed proglottids and consequently were considered to be infected with *D. caninum*. The first proglottids to be detected were present in the faeces of two cats in the untreated control group on Day 26. The majority of untreated cats and the infected treated cat had started shedding proglottids by Day 35, but one cat only started shedding proglottids on Day 46 and another on Day 51.

**Table 4:** **The presence of*Dipylidium caninum*proglottids in the faeces and surroundings of untreated cats and of cats fitted with imidacloprid/flumethrin collars**

**Untreated control cats**		**Cats fitted with collars**	
**Cat ID**	**Days proglottids detected**	**Cat ID**	**Days proglottids detected**
CAE	26 and 27	441	None
E9D	26 and 27	844	None
AD3	29 and 31	8D3	None
E38	34 and 35	FAC	34 and 35
34D	34 and 37	680	None
AD1	35 and 36	622	None
B53	46 and 49	6AA	None
296	51 and 52	586	None
**Infection (%)**	**100**	**Infection (%)**	**12.5**

The numbers of *D. caninum* scoleces recovered from the cats at necropsy are recorded in Table 5. All the cats in the untreated control group were infected with *D. caninum*, and the number of scoleces collected from individual animals varied between 19 and 346. Only one of the cats in the collared group was infected with *D. caninum* and it harboured 2 scoleces. It was also the only cat in the collared group to shed proglottids. The geometric mean number of scoleces recovered from the untreated control group of cats differed significantly (P < 0.05) from the number recovered from the collared group of animals. Based on a comparison of the geometric mean number of scoleces recovered from the control and treated cats, the imidacloprid/flumethrin collars were 99.7% effective in preventing *D. caninum* infection in cats infested with infected fleas.

**Table 5:** ***Dipylidium caninum*scoleces recovered from untreated control cats and from cats fitted with imidacloprid/flumethrin collars**

**Untreated control cats**		**Cats fitted with medicated collars**	
**Cat ID**	**Number of scoleces**	**Cat ID**	**Number of scoleces**
CAE	116	441	0
E9D	91	844	0
AD3	346	8D3	0
E38	20	FAC	2
34D	88	680	0
AD1	19	622	0
B53	28	6AA	0
296	37	586	0
Total	745	Total	2
Geometric mean	58.3	Geometric mean	0.1
		**Prophylaxis (%)**	**99.7**

## Discussion

The efficacy or 99.9% to 100% of the imidacloprid/flumethrin collar (Seresto ^©^) against repeated infestations with *C. felis felis* during the first 28 days of the study confirms the results of earlier investigations on the efficacy of the collars against fleas on cats [11]. Despite the fact that fleas that had been counted were released back onto the same cat from which they had been collected, no increase in flea numbers was noted on the untreated cats. In fact the greatest mean number of fleas (76.8) was collected from the cats infested for the first time on Day 7, whereas the mean numbers of fleas recovered from the same cats infested on subsequent weekly occasions and on which fleas had been released after counting, remained below 75. The absence of an increase in flea numbers can be ascribed to the extraordinary grooming efficiency of cats. For instance Hinkle *et al*. [12] reported that a cat that was a good groomer removed up to 17.6% of its flea burden daily. A poor groomer on the other hand removed only 4.1% of its flea burden daily. They also reported that the mean longevity of fleas on the four artificially infested cats in their study was only 7.8 days.

Although differences in the faecal worm egg counts between treated and control groups of animals may in some instances prove to be valid for the determination of anthelmintic efficacy against nematodes, no such method exists for efficacy studies against cestodes. The shedding of *D. caninum* proglottids is neither quantitative nor consistent. This phenomenon is evident for three cats in the untreated control group, for which there was a break of 2 to 3 days between the first and the following appearance of proglottids in their faeces or surroundings, and two cats that only started shedding proglottids 46 and 51 days into the study (Table 4).

The first proglottids of *D. caninum* to be shed by the cats were detected 26 days after the commencement of the study, or 19 days after infestation with the first batch of infected fleas on Day 7. The prepatent period of *D. caninum* was thus 19 days or perhaps less, depending on the day on which the infected flea or fleas were swallowed. Prepatent periods of 17 and of 23 days have been reported for *D. caninum* in cats that were fed infected fleas [8], and the prepatent period of 19 days is thus in agreement with those findings. The proglottids shed by cats can remain infective for flea larvae for several weeks or months depending on temperature [8] and provided that there is sufficient moisture to prevent the eggs from drying out [13].

Early researchers believed that the development of metacestodes in their flea hosts was linked to the development of the flea from larva to adult [14]. Subsequent research by Pugh [4] and Pugh and Moorhouse [15] has, however, demonstrated that development is controlled by temperature. They found that at a temperature of 20°C no perceptible growth of metacestodes occurs in the flea host [4], whereas at temperatures between 20 and 25°C development is slow and the flea host’s reaction to infection more intense and sustained than at higher temperatures [4]. The decrease in the prevalence of infection with metacestodes as the age of the infected fleas in the present study increased (Table 3) can possibly be ascribed to two factors. The first being that the fleas had mounted a successful response to infection and that the metacestodes had degenerated [4]. This phenomenon was, however, not observed in the 800 fleas that were dissected. The second and more likely occurrence was that infected, unfed adult fleas died more rapidly than their uninfected counterparts.

In his studies Pugh [4] noted that at temperatures below 30°C metacestodes were unable to complete their development in adult fleas unless the fleas were placed on a mammalian host for a few days. The host’s surface temperature, and not blood meals taken by the flea, then enabled the metacestodes to mature and become infective for the definitive host [4]. The latter observation is of particular significance in the present study during which the fleas in the pool used for infestation were maintained at temperatures ranging from 24°C to 28.5°C. At this temperature range the majority of metacestodes in the fleas in the 7 to 22-day old groups had not fully developed (Table 3) and many would thus have required that their flea hosts infested a mammalian host before they became infective.

It was precisely for this reason that once fleas had been counted they were released back onto the same cat from which they had come, so as to ensure that any metacestodes with which they were infected could develop to maturity and thus more closely resemble conditions pertaining in the field. Infection with *D. caninum* in the single cat in the collared group suggests that some metacestodes may complete their development off-host at temperatures below 30°C. It is thus possible that the latter animal had been infested with a 14-day old flea or fleas in which the metacestodes had already fully developed, and this flea(s) was ingested shortly after release onto the cat.

Within a home environment the temperature may fluctuate considerably from night to day, but the average daily temperature is unlikely to exceed 25°C. In barns, stables, and outside rooms or on verandahs frequented by cats the average daily temperature is liable to be even lower. Under these circumstances infected fleas would have to spend a few days on a cat before the metacestodes become infective. It is during this pre-infective period on the host that infected fleas must be killed to prevent infection of the host animal with *D. caninum*. Studies prior to the present one have shown that imidacloprid/flumethrin collars killed >99% of fleas that access a cat within 24 h after the collars had been applied [11]. Furthermore the collars also proved to be >95% effective within 24 h of subsequent infestations with *C. felis felis* for a period of 8 months [11]. It is thus the rapidity with which the active ingredients of the collars kill fleas that prevents them spending sufficient time on the host for the metacestodes with which they may be infected to develop to maturity and able to infect a host.

In a field situation, cat owners who detect fleas on their animals and who also notice single or strings of proglottids in the cats’ faeces or hair coat are likely to seek advice from their local veterinarians. The animals will then be treated for tapeworms and the owners informed that fleas must be controlled on the cats. Should imidacloprid/flumethrin collars be used for flea control in this scenario the prolonged period of protection they afford against flea infestation will play an essential role in the prevention of re-infection with *D. caninum.* The reasoning behind this is that flea larvae, and hence adult fleas, that were already present in the cats’ environment before treatment, are likely to become infected with metacestodes originating from *D. caninum* proglottids that had been shed several weeks or even months previously [8], and that these infected fleas only access the cats after the collars have been fitted.

Not only are the imidacloprid/flumethrin collars effective against adult fleas, but they are also effective against flea larvae in the cats’ immediate surroundings [11]. Consequently if larvae are eliminated in the collared cat’s favourite sleeping, resting or loafing places in the home or beyond its confines, the numbers available to ingest *D. caninum* eggs will be drastically reduced. This in turn will result in a significant decline in the number of infected adult fleas, of which the vast majority will in turn be killed on the cat.

## Conclusion

The speed with which the insecticidal components of 10% imidacloprid/4.5% flumethrin collars eliminate newly-acquired infestations of fleas that are infected with the metacestodes of *D. caninum*, and their sustained high level of efficacy, indicate that infection of cats with this cestode can be prevented by application of the collars.

## Competing interests

This clinical study was completely funded by Bayer Animal Health GmbH, Monheim, Germany, of which D. Stanneck (Germany) is an employee. ClinVet, of which J.J. Fourie and D. Crafford are employees, is an independent, South African, Contract Research Organisation contracted to manage the conduct of the study. I.G. Horak is a long-term, contract employee of Clinvet and an Extraordinary Professor at the Universities of the Free State and Pretoria. All authors voluntarily publish this article and have no personal interest in these studies other than publishing the scientific findings that they have been involved in via planning, initiating, monitoring and conducting the investigations and analysing the results.

## Author’s contributions

DS and JJF designed the study and drafted the protocol and JJF conducted the study with the assistance of DC who dissected the fleas and diagnosed infection in the cats. IGH collated and organized the data and compiled the initial draft of the manuscript. All authors read and approved the final manuscript.

## References

[B1] Beaucournu JC Ménier K Le genre Ctenocephalides Stiles et Collins, 1930 (Siphonaptera, Pulicidae) Parasite 1998 5 3 16 9754292 10.1051/parasite/1998051003

[B2] Ménier K Beaucournu JC Approche biogeographique du genre Ctenocephalides Stiles et Collins, 1930 (Insecta: Siphonaptera) Biographica 1999 75 79 88

[B3] Genchi C Traldi G Bianciardi P Efficacy of imidacloprid on dogs and cats with natural infestations of fleas, with special emphasis on flea hypersensitivity Vet Ther 2000 1 71 80 19757553

[B4] Pugh RE Effects on the development of Dipylidium caninum and on the host reaction to this parasite in the adult flea (Ctenocephalides felis felis) Parasitol Res 1987 73 171 177 10.1007/BF00536475 3575292

[B5] Ash LR Helminth parasites of dogs and cats in Hawaii J Parasitol 1961 48 63 65 13862798

[B6] Wilson-Hanson SL Prescott CW A survey for parasites in cats Aust Vet J 1982 59 194 10.1111/j.1751-0813.1982.tb16009.x 7168726

[B7] Baker MK Lange L Verster A Van der Plaat S A survey of helminths in domestic cats in the Pretoria area of Transvaal, Republic of South Africa. Part 1: The prevalence and comparison of burdens of helminths in adult and juvenile cats J S Afr Vet Ass 1989 60 139 142 2634770

[B8] Boreham RE Boreham PFL Dipylidium caninum: life cycle, epizootiology, and control Compendium 1990 12 667 675

[B9] Moore DV A review of human infections with the common dog tapeworm, Dipylidium caninum, in the United States Southwestern Vet 1962 283 288 Summer

[B10] Chappell CL Pen HM Dipylidium caninum, an underrecognized infection in infants and children Pediatric Infectious Dis J 1990 9 745 747 10.1097/00006454-199010000-00014 2235150

[B11] Stanneck D Kruedewagen EM Fourie JJ Horak IG Davis W Krieger KJ The efficacy of an imidacloprid/flumethrin collar against fleas and ticks on cats Par Vect 2012 5 82 10.1186/1756-3305-5-82 PMC3434012 22541037

[B12] Hinkle NC Koehler PG Patterson RS Host grooming efficiency for regulation of cat flea (Siphonaptera: Pulicidae) populations J Med Entomol 1998 35 266 269 9615545 10.1093/jmedent/35.3.266

[B13] Venard CE Morphology, bionomics and taxonomy of the cestode Dipylidium caninum Anns N Y Acad Sci 1938 1938 37 273 328

[B14] Marshall AG The cat flea, Ctenocephalides felis felis (Bouché, 1835) as an intermediate host for cestodes Parasitology 1967 57 419 430 10.1017/S0031182000072322 6048565

[B15] Pugh RE Moorhouse DE Factors affecting the development of Dipylidium caninum in Ctenocephalides felis felis (Bouché, 1835) Z Parasitenkd 1985 71 765 775 10.1007/BF00926802 4082734

